# Automated assessment of balance function based on a lightweight end-to-end multimodal deep learning framework: predicting Mini-BESTest scores from center of pressure signals in a single stance condition

**DOI:** 10.3389/fbioe.2026.1936471

**Published:** 2026-09-24

**Authors:** Yuyi Fan, Yunfei Gui, Linyan Liu, Linlin Zhang, Yi Yu, Roger Adams, Jia Han, Jinfeng Cao, Jie Lyu

**Affiliations:** 1 School of Health Science and Engineering, University of Shanghai for Science and Technology, Shanghai, China; 2 College of Rehabilitation Sciences, Shanghai University of Medicine and Health Sciences, Shanghai, China; 3 College of Medical Instruments, Shanghai University of Medicine and Health Sciences, Shanghai, China; 4 Research Institute for Sport and Exercise, University of Canberra, Canberra, ACT, Australia; 5 Periodicals Agency of Shanghai University, Shanghai University, Shanghai, China; 6 Department of Orthopedics, Jinshan District Central Hospital Affiliated to Shanghai University of Medicine and Health Sciences, Shanghai, China

**Keywords:** balance assessment, center of pressure, deep learning, end-to-end learning, lightweight neural network, mini-bestest, multimodal fusion

## Abstract

**Background:**

Balance dysfunction is a common health issue among older adults and patients with neurological disorders. The Mini-Balance Evaluation Systems Test (Mini-BESTest) is a widely used clinical tool for balance assessment. However, its full implementation requires a trained clinician to administer 14 tasks, and the scoring is subject to inter-rater variability.

**Objective:**

This study aims to develop an end-to-end, lightweight multimodal deep learning framework (with approximately 55 K parameters) to automatically predict the total Mini-BESTest score and its four subscale scores, utilizing only center of pressure (COP) signals from a single stance condition alongside clinical metadata.

**Methods:**

COP signals and clinical metadata from 163 subjects in a public dataset (Santos et al., PeerJ, 2016, 4) were utilized. We proposed a lightweight multimodal deep learning model comprising three dilated convolution branches with varying dilation rates, a channel attention mechanism, and a metadata fusion module. Three independent comparative experiments were conducted to investigate the effects of stance condition, trial number, and time window length on predictive performance. Based on these results, a suitable data configuration was selected for subsequent experiments. An adaptive task weighting scheme based on homoscedastic uncertainty was employed to balance the five regression targets with varying score ranges. Five-fold cross-validation was used to evaluate the models, and out-of-fold (OOF) predictions were constructed to provide a full-sample assessment of predictive performance.

**Results:**

Under the selected data configuration (foam surface with eyes open, second trial, first 30 s of data), the five-fold cross-validation mean absolute error (MAE) for the predicted Mini-BESTest total score was 1.971 (out of 28). The full-sample OOF prediction MAE was 1.979, with a Pearson correlation coefficient of r = 0.758 and R^2^ = 0.574. In ablation experiments, removing the metadata branch led to the largest numerical increase in total-score MAE (to 2.338), while removal of the channel attention module resulted in a smaller increase (to 2.034). A metadata-only model achieved a total-score MAE of 2.105. Paired Wilcoxon signed-rank tests did not show statistically significant differences between the full model and the ablated variants (all p > 0.05).

**Conclusion:**

This study demonstrates that an end-to-end deep learning framework can predict the complete Mini-BESTest score from 30 s of quiet standing COP signals under foam surface eyes-open conditions, together with basic clinical metadata. While maintaining predictive accuracy, this lightweight framework (approx. 55 K parameters) may represent a potential direction for future deployment in portable or remote settings, subject to further engineering and clinical validation.

## 1 Introduction

Balance control is fundamental for maintaining activities of daily living and involves the coordinated operation of multiple subsystems, including anticipatory postural adjustments, reactive postural control, sensory orientation, and dynamic gait (Franchignoni et al., 2010). Balance dysfunction is prevalent among older adults and patients with neurological disorders, serving as a primary cause of falls and related injuries (Ganz and Latham, 2020). It is reported that approximately one-quarter of community-dwelling adults over 65 experience at least one fall annually, some resulting in injuries requiring medical attention (Burns and Kakara, 2018). Therefore, accurate and objective assessment of balance function is of significant importance for fall risk screening, formulation of rehabilitation interventions, and monitoring of treatment efficacy.

The center of pressure (COP) is a core metric measured by force plates, defined as the point of application of the ground reaction force on the support surface. It has been utilized in postural control research for decades (Scoppa et al., 2013). Traditional analytical methods for COP signals encompass time-domain analysis (e.g., root mean square, sway area, path length), frequency-domain analysis (e.g., power spectral density, mean power frequency), and nonlinear analysis (e.g., sample entropy, multiscale entropy) (Palmieri et al., 2002; Gow et al., 2015). However, variations in data acquisition parameters (e.g., sampling frequency, test duration, stance posture standardization) and COP feature calculation methods across studies may compromise inter-study comparability (Ruhe et al., 2011). Additionally, COP signals inherently exhibit non-stationary characteristics, presenting limitations for traditional Fourier analysis in capturing dynamic changes (Pascolo et al., 2006). Feature engineering generally requires considerable domain knowledge and may omit information pertinent to balance control (Duarte and Freitas, 2010). Furthermore, single COP parameters often fail to correspond directly to specific balance subsystem functions (Mancini and Horak, 2010).

The Mini-BESTest is a widely utilized clinical balance assessment tool, condensed from the original 36-item BESTest by Franchignoni et al., in 2010 via factor analysis and the Rasch model (Franchignoni et al., 2010). It comprises 14 task items evaluating four postural control subsystems: Anticipatory Postural Adjustments (APA, 0–6 points), Reactive Postural Control (RPC, 0–6 points), Sensory Orientation (SO, 0–6 points), and Dynamic Gait (DG, 0–10 points), yielding a maximum total score of 28 (Horak et al., 2009). The Mini-BESTest has demonstrated robust reliability and validity across diverse populations, including individuals with stroke (Tsang et al., 2013), Parkinson’s disease (Di Carlo et al., 2016), spinal cord injury (Roy et al., 2021; Morooka et al., 2024), and healthy older adults (Yingyongyudha et al., 2016). Previous studies indicate an excellent test-retest reliability (ICC = 0.90–0.97) (Tsang et al., 2013; Huang et al., 2016) and high internal consistency (Cronbach’s α = 0.89–0.94) (Tsang et al., 2013).

Despite its clinical utility, Mini-BESTest scoring exhibits inherent inter-rater variability, as different evaluators may render inconsistent judgments (Sibley et al., 2015). Moreover, full administration of the scale requires the physical presence of a trained clinician, limiting its widespread application in scenarios lacking specialized personnel, such as community screening and remote rehabilitation monitoring. Consequently, developing a computational method capable of automatically generating balance assessment scores based on objective physiological signals holds potential value for expanding the accessibility of balance evaluation.

In recent years, deep learning technologies have demonstrated promise in the field of balance assessment. Previous work utilizing the same public dataset extracted 224 handcrafted features and applied traditional regressors (e.g., Random Forest, MLP) to predict Mini-BESTest scores, achieving a Mean Absolute Error (MAE) of approximately 2.658 for the total score (Ren et al., 2020). Choi et al. (2024) employed a Convolutional Neural Network (CNN) model to predict Sensory Organization Test balance scores from frequency-domain COP features, yielding an absolute difference of roughly 1.7 (Choi et al., 2024). Other studies have also explored deep architectures, such as CNNs and LSTMs, for processing COP signals or force plate data (Phan and Lee, 2022; Duangnga et al., 2026). Nevertheless, current research predominantly relies on handcrafted feature extraction, leaving room for exploration in automatically learning features directly from raw COP signals.

Furthermore, multimodal fusion is increasingly applied in medical signal processing. Evidence suggests that integrating clinical metadata (e.g., age, cognitive function assessments, fear of falling) with physiological signals can enhance predictive model performance (Caronni et al., 2023; Jaén-Vargas et al., 2025). To date, research utilizing multimodal fusion of COP signals and clinical metadata to predict Mini-BESTest scores remains limited. Beyond force-plate-based analyses, recent work has also explored technology-enabled functional assessment using video-based approaches, such as dual-task mobility testing for fall-risk evaluation in neurological populations (Aytutuldu et al., 2026). Lightweight deep learning models have garnered attention due to their suitability for edge device deployment (Iqbal et al., 2024). While lightweight models have been developed for ECG and EEG signals in the medical domain (Baig et al., 2026), architectures specifically designed for COP signals are scarce.

In this study, the problem was formulated as a multi-output regression task: given a 30-s COP recording from a single stance condition and a set of clinical metadata, the model learns a direct mapping to the Mini-BESTest total score and its four subsystem scores. Based on the aforementioned context and using a publicly available community-mixed balance dataset, the contributions of this study are as follows.

1. We propose an end-to-end multimodal deep learning framework that predicts the Mini-BESTest total score and its four subscale scores directly from raw COP signals and clinical metadata, without handcrafted feature engineering.
2. We demonstrate that a 30-s quiet-standing recording from a single condition (foam surface, eyes open) is sufficient for this prediction within the present dataset, thereby simplifying the data collection procedure compared with approaches that require multiple standing conditions.
3. We design a lightweight model architecture (approximately 55 K parameters) that maintains predictive accuracy and may be suitable for future deployment in resource-constrained settings.
4. We perform ablation studies and include a simple linear regression baseline, suggesting that COP signals and clinical metadata may provide complementary information.

## 2 Methods

### 2.1 Dataset

This study utilized a publicly available human balance evaluation dataset published by Santos and Duarte (2016), accessible via PhysioNet. The dataset comprises 163 subjects (116 females, 47 males, age range 18–85 years) who performed 60-s quiet standing tests under four different conditions: rigid surface with eyes open (Rigid + EO), rigid surface with eyes closed (Rigid + EC), foam surface with eyes open (Foam + EO), and foam surface with eyes closed (Foam + EC). Each condition was repeated three times in a randomized order. COP signals were acquired using an AMTI OPT400600-1000 force plate at a sampling frequency of 100 Hz.

In addition to COP signals, the dataset contains clinical assessment information for each subject, including the 14-item scores of the Mini-BESTest, Short Falls Efficacy Scale International (Short FES-I), International Physical Activity Questionnaire Short Version (IPAQ-SV), and the Trail Making Test (TMT), as well as demographic (age, gender, height, weight, BMI) and health-related information. Clinical metadata (such as age, FES, and TMT) are routine clinical assessment parameters typically collected during initial patient visits or annual physical examinations and can be directly retrieved from electronic health records.

Five subjects failed to complete all trials (particularly under the Foam + EC condition), resulting in slight variations in the actual number of subjects across conditions (158–163). For subjects with missing trials under a specific condition, the mean predicted value from the available trials was used as the final prediction, without imputation or exclusion.

### 2.2 Calculation of target variables

The Mini-BESTest consists of 14 items (Best_1 to Best_14), among which item 3 (single-leg stance) and item 6 (compensatory stepping) involve bilateral testing. According to the official scoring guidelines (Franchignoni et al., 2010), the lower score between the left and right sides is taken as the final score for that item. The four subsystems and the total score were calculated as follows.

- APA = Best_1 + Best_2 + min (Best_3L, Best_3r)
- RPC = Best_4 + Best_5 + min (Best_6L, Best_6r)
- SO = Best_7 + Best_8 + Best_9
- DG = Best_10 + Best_11 + Best_12 + Best_13 + Best_14
- TOTAL = APA + RPC + SO + DG

This calculation method has been verified to perfectly align with the Best_T field provided in the dataset.

### 2.3 Data preprocessing

COP signals were initially subjected to baseline correction: the mean value of the first 10 sampling points (0.1 s) was taken as the baseline, and this value was subtracted from the entire time series. This eliminates individual differences in the initial standing position and centers the COP signal around zero. Baseline correction is a standard preprocessing step in postural control research.

During each fold of the cross-validation, COP signals in the training set were standardized using the StandardScaler (z-score standardization fitted on the training set and applied to the validation set). Numerical metadata were similarly standardized via z-score scaling within each fold. Categorical features were processed using One-Hot encoding (retaining all dummy variable dimensions) without standardization. The target variables were standardized prior to training and inversely transformed to their original scale after prediction.

### 2.4 Input features

The input to the model comprises two components:

COP Signal: A 2-channel array (anterior-posterior COPx and medio-lateral COPy) × sequence length. Based on the results of three comparative experiments, the final selected data configuration was the first 30 s of data from the second trial under the Foam + EO condition (3,000 sampling points, 100 Hz).

Clinical Metadata: Includes eight continuous numerical features (age, height, weight, BMI, foot length, TMT_timeA, TMT_timeB, FES_T) and four categorical features (gender, age group, FES_S, IPAQ_S). Categorical features were One-Hot encoded, retaining all dimensions.

### 2.5 Model architecture

The proposed model is an end-to-end, lightweight multimodal deep learning architecture with approximately 55 K parameters. The model consists of two parallel feature extraction branches processing the COP signal and clinical metadata separately, which are subsequently merged via a fusion layer to output five predicted scores. Detailed parameters for each layer are provided in Table 1, and the parameter distribution across modules is shown in Table 2. A comprehensive schematic of the proposed multimodal architecture is illustrated in Figure 1.

**TABLE 1:** Layer-wise parameters of the model.

Layer name	Input shape	Output shape	Parameter description
Dilated conv (d = 1)	(2, T)	(32, T)	kernel = 3, padding = 1, dilation = 1
Dilated conv (d = 4)	(2, T)	(32, T)	kernel = 3, padding = 4, dilation = 4
Dilated conv (d = 16)	(2, T)	(32, T)	kernel = 3, padding = 16, dilation = 16
Concatenate	3×(32, T)	(96, T)	Concatenation along channel dimension
Channel attention	(96, T)	(96, T)	GAP→FC(96→24)→ReLU→FC(24→96)→Sigmoid
Downsample conv	(96, T)	(64, T/2)	kernel = 5, stride = 2, padding = 2
Max pooling	(64, T/2)	(64, T/4)	kernel = 2, stride = 2
Global avg pooling	(64, T/4)	(64)	Adaptive global average pooling
Meta MLP layer 1	(meta_dim)	(64)	FC→ReLU→Dropout (0.1)
Meta MLP layer 2	(64)	(32)	FC→ReLU
Fusion layer 1	(96)	(32)	FC→ReLU→Dropout (0.2)
Output layer	(32)	(5)	FC

T represents the sequence length of the input; meta_dim represents the dimensionality of the metadata (20 for a single condition).

**TABLE 2:** Core network architecture and parameter distribution.

Module	Description	Number of parameters
COP branch (temporal features)	Dilated conv (d = 1,4,16) + attention + pooling	48,736
Metadata branch (clinical info)	Meta MLP	3,040
Feature fusion & output layer	Fusion FC(Multi-task output)	3,269
Total model parameters	Total trainable parameters	55,045

**FIGURE 1:**
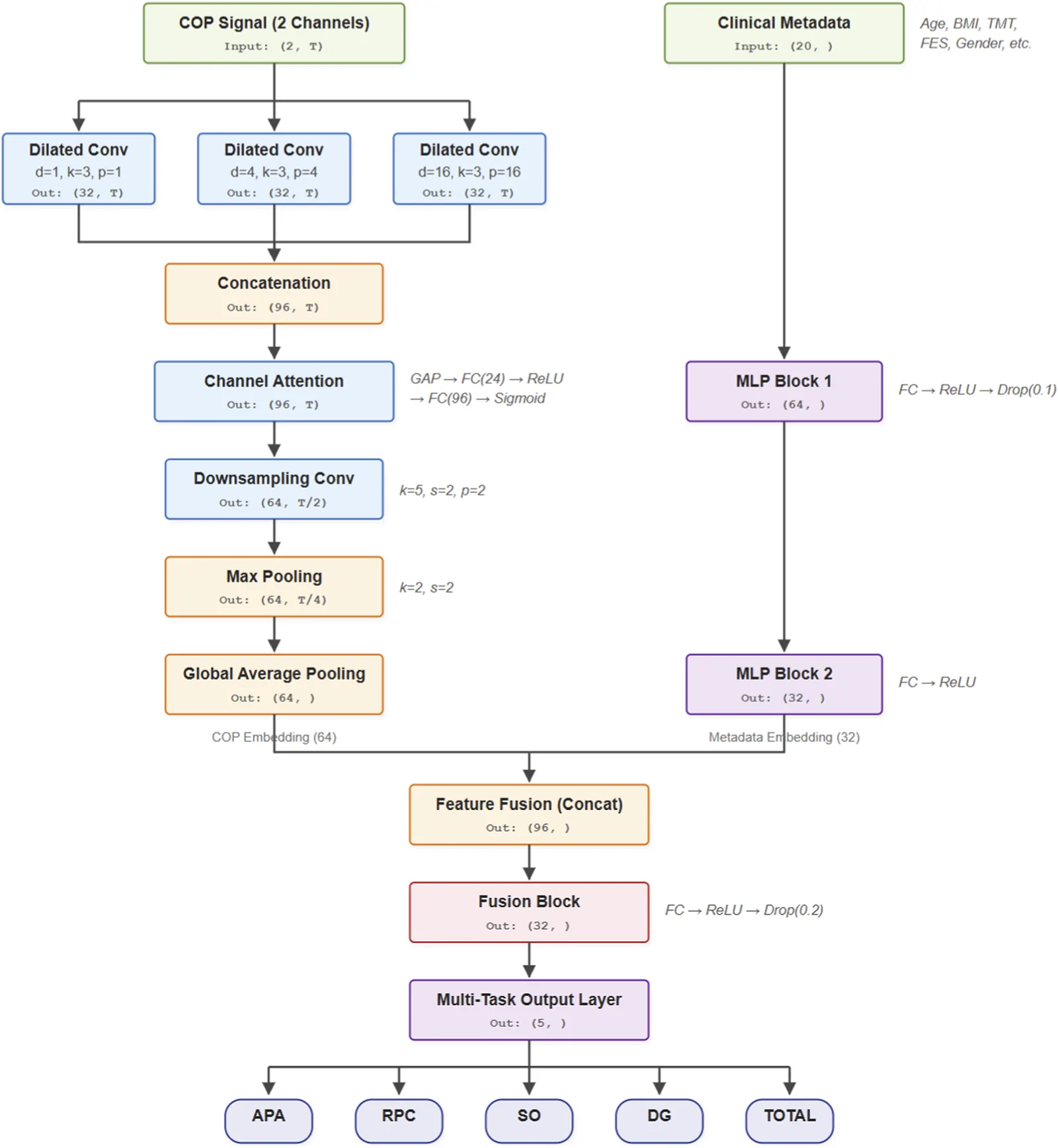
Schematic diagram of the model architecture.

COP Branch: The raw COP signal (shape 2 × T, where two denotes the anterior-posterior and medio-lateral channels, and T is the sequence length) is passed in parallel through three Dilated Convolution blocks. Each block shares the same structural design but utilizes different dilation rates (d = 1, d = 4, and d = 16). These varying dilation rates correspond to different effective receptive field sizes (capturing varying temporal scales at a 100 Hz sampling rate), effectively covering features ranging from rapid postural reactions to slower postural drifts. Each block contains a 1D convolutional layer with a kernel size of three (input channels = 2, output channels = 32, padding = d to preserve sequence length), a Batch Normalization layer, and a ReLU activation function. The outputs of the three branches are concatenated along the channel dimension to form a 96-channel feature map.

Channel Attention: The concatenated feature map is fed into a Channel Attention module. This module first performs global average pooling to compress the spatial information of each channel into a single scalar. Subsequently, a bottleneck structure comprising two fully connected layers (96→24→96, compression ratio = 4) is applied, utilizing ReLU activation after the first layer and Sigmoid activation after the second, generating a 96-dimensional channel weight vector. This weight vector is multiplied element-wise with the original feature map to achieve adaptive recalibration of the channel features.

Downsampling Module: The recalibrated feature map is processed sequentially through a downsampling module. This consists of a 1D convolutional layer (kernel size = 5, stride = 2, padding = 2, input channels = 96, output channels = 64) that halves the sequence length, followed by a ReLU activation. A Max Pooling layer (window size = 2) further halves the sequence length. Finally, an Adaptive Global Average Pooling layer compresses the variable-length sequence features into a fixed-length 64-dimensional COP feature vector.

Metadata Branch: The clinical metadata vector (comprising eight standardized continuous numerical features and One-Hot encoded categorical features) is encoded via a two-layer fully connected network. The first layer projects the metadata dimension to 64 dimensions, followed by a ReLU activation and Dropout (rate = 0.1). The second layer maps the 64 dimensions to 32 dimensions, followed by a ReLU activation.

Fusion and Output: The COP feature vector (64-dimensional) and the metadata feature vector (32-dimensional) are concatenated along the feature dimension to form a 96-dimensional joint feature vector. This vector is passed through a fusion layer, initially mapped to 32 dimensions with ReLU activation and Dropout (rate = 0.2), and subsequently mapped by the output layer to five dimensions, corresponding to the predicted scores for APA, RPC, SO, DG, and TOTAL.

### 2.6 Loss function

The five prediction targets of the Mini-BESTest have varying score ranges (APA: 0–6, RPC: 0–6, SO: 0–6, DG: 0–10, TOTAL: 0–28). To balance the learning process across different tasks, this study adopted an adaptive task weighting scheme based on homoscedastic uncertainty (Cipolla et al., 2018). This approach models the loss for each task as:

$$L=\sum_{i}\left(\frac{1}{2\sigma_{i}^{2}}\cdot {\text{MSE}}_{i}+\log\sigma_{i}\right)$$

where $\sigma_{i}^{2}$ is a learnable task-specific noise parameter automatically optimized during model training. The initial value of $\sigma_{i}$ was set to 1.0 and updated synchronously with other model parameters via gradient descent. Under this framework, tasks with larger noise (i.e., tasks harder to fit) automatically receive lower weights, whereas tasks with smaller noise receive higher weights. This method avoids the need to manually pre-define task weights and offers broad applicability.

### 2.7 Experimental design

Three sets of comparative experiments were designed to investigate the impact of stance conditions, trial numbers, and time window lengths on model predictive performance (Experiments one to three). Each experiment was conducted independently using full five-fold cross-validation. The outcomes were utilized to observe the relationship between these factors and prediction accuracy, ultimately serving to select a suitable data configuration for subsequent analyses.

Experiment 1 (Selection of Stance Conditions): The full 60-s data were utilized under four single conditions (Rigid/Foam surface × Eyes Open/Closed) and a mixture of all conditions (All Conditions). Each trial from a subject was treated as an independent sample during training. During evaluation, the predictions for all trials of the same subject were averaged and compared against their true Mini-BESTest scores. Based on the results, a condition demonstrating balanced comprehensive performance and high data completeness was selected.

Experiment 2 (Selection of Trial Number): Under the condition selected in Experiment 1, the first, second, and third trials were utilized independently for training and evaluation, and compared against the performance of averaging predictions across all three trials. The trial exhibiting relatively lower prediction error was selected for subsequent experiments.

Experiment 3 (Selection of Time Window): Under the configurations determined in Experiments 1 and 2, COP data corresponding to six time windows ranging from 10 s to 60 s (in 10-s increments) were used for training and evaluation. Based on the trade-off between accuracy and data acquisition efficiency, a suitable time window was selected.

Using the data configuration established from the above experiments, an ablation study (Full Model vs. Removed Metadata Branch vs. Removed Channel Attention Module) was conducted to evaluate the contribution of each component. Following the finalization of the data configuration, full five-fold cross-validation was run independently for each ablation variant and for final performance evaluation.

In addition, a simple linear regression baseline was evaluated using only the clinical metadata features. This baseline was trained and tested under the same subject-level five-fold cross-validation protocol to provide a reference for the performance of the proposed deep learning model.

### 2.8 Training and evaluation

All experiments utilized five-fold stratified cross-validation based on subject splits (ensuring data from the same subject did not appear simultaneously in the training and validation sets). Within each fold, all preprocessing steps—including standardization of COP signals and numerical metadata, one-hot encoding of categorical features, and any averaging across trials—were derived exclusively from the training subjects. No imputation was performed for missing trials. For subjects with fewer than three available trials in a given condition, predictions were based on the available trials only; subjects with no available trials for a condition were excluded from analyses involving that condition. The median of the total score was used as the stratification criterion to ensure that the proportion of high-score and low-score subjects in each validation fold remained approximately consistent with the overall dataset.

The model was trained using the AdamW optimizer (learning rate = 1e-3) for 100 epochs, with a batch size of 32. Mixed precision training and gradient clipping (max_norm = 1.0) were employed to stabilize training. Hyperparameter selection adhered to standard practices for small-sample physiological time-series data without extensive grid searches, avoiding overfitting to the validation set.

To obtain an estimate across the entire sample and to support visualization, an OOF strategy was implemented. Specifically, within each fold of the cross-validation, the model fitted on the training set was used to generate predictions for every subject in that fold’s validation set. Since each subject was assigned to a validation set exactly once, concatenating the predicted values across all five folds yielded a complete full-sample OOF prediction set (n = 160). All subsequent evaluation metrics (MAE, Pearson’s r, coefficient of determination R^2^) and scatter plots strictly rely on this OOF result set, ensuring consistency between the reported values and the visualized predictions.

It should be noted that Experiments one to three were designed to identify suitable data input methods by comparing predictive performance across configurations. Given the limited sample size (163 subjects), a separate hold-out validation set was not allocated for configuration screening; instead, complete five-fold cross-validation was used throughout. Therefore, the predictive performance reported herein should be interpreted as an internal validation estimation on this dataset, and its generalization to completely independent external data requires further verification.

### 2.9 Evaluation metrics

The primary evaluation metric was the Mean Absolute Error (MAE). Results are reported in the format of the mean MAE across the five folds ± standard deviation (SD), reflecting the model’s performance fluctuation under different training/validation splits. A Bootstrap method (1,000 iterations of resampling with replacement) was utilized to estimate the 95% Confidence Interval (CI) of the MAE. This method was based on the OOF absolute errors of all subjects to indicate the precision of the overall MAE estimation. For high-density data presentation, all tables display data uniformly in the format of MAE ± SD [95% CI Lower Limit, 95% CI Upper Limit]. Additionally, the Pearson correlation coefficient (r) and coefficient of determination (R2) were reported to assess linear consistency between predicted and ground-truth values.

## 3 Results

### 3.1 Selection of Stance Conditions

Table 3 presents the predictive performance of the model under four single stance conditions and a mixture of all conditions. The Foam surface conditions (Foam + EO and Foam + EC) achieved lower MAE in the total score and most subsystems compared to the Rigid surface conditions. Mixing all conditions yielded relatively poorer performance (TOTAL MAE = 2.300), suggesting that COP patterns might differ considerably across stance conditions, and mixed training may introduce additional variance.

**TABLE 3:** Predictive performance under different stance conditions (60s, averaged across all trials, MAE ± SD [95% CI]).

Condition	n	APA	RPC	SO	DG	TOTAL
Rigid + EO	163	0.753 ± 0.110 [0.659, 0.851]	1.043 ± 0.056 [0.924, 1.172]	0.728 ± 0.102 [0.637, 0.828]	1.027 ± 0.133 [0.913, 1.141]	2.200 ± 0.214 [1.868, 2.571]
Rigid + EC	163	0.756 ± 0.115 [0.682, 0.841]	1.021 ± 0.037 [0.897, 1.160]	0.722 ± 0.087 [0.621, 0.824]	1.005 ± 0.136 [0.899, 1.115]	2.209 ± 0.312 [1.881, 2.576]
Foam + EO	160	0.695 ± 0.057 [0.606, 0.787]	1.024 ± 0.183 [0.894, 1.153]	0.651 ± 0.081 [0.567, 0.738]	0.976 ± 0.045 [0.866, 1.088]	2.078 ± 0.337 [1.776, 2.415]
Foam + EC	158	0.704 ± 0.065 [0.618, 0.794]	0.942 ± 0.148 [0.831, 1.049]	0.652 ± 0.040 [0.570, 0.745]	0.975 ± 0.060 [0.873, 1.082]	2.021 ± 0.338 [1.744, 2.321]
All conditions	163	0.752 ± 0.084 [0.663, 0.848]	1.030 ± 0.074 [0.905, 1.167]	0.712 ± 0.069 [0.617, 0.809]	1.043 ± 0.140 [0.936, 1.163]	2.300 ± 0.224 [1.972, 2.694]

n = number of subjects; MAE, mean MAE across five-fold cross-validation; SD, standard deviation of five-fold MAE; CI, Bootstrap 95% confidence interval (1,000 resamples).

Considering predictive accuracy, data completeness (160 subjects for Foam + EO, 158 for Foam + EC), and clinical feasibility (eyes-open conditions are generally easier to standardize than eyes-closed), the Foam + EO condition was selected for subsequent experiments.

### 3.2 Selection of Trial Number

Table 4 displays the predictive performance of different trial strategies under the Foam + EO condition. Utilizing solely the second trial (Trial 2) yielded the lowest prediction error (TOTAL MAE = 1.989), followed by the third trial (2.018) and the first trial (2.080). Averaging across all three trials produced a TOTAL MAE of 2.078 (Table 3), which was higher than using Trial two alone. This indicates that the first trial may be influenced by unfamiliarity with the task, and the third trial by fatigue, suggesting that mixing the three could introduce variance. Consequently, Trial two was adopted as the standard input for subsequent experiments.

**TABLE 4:** Predictive performance under different trial strategies (foam + EO, 60s, MAE ± SD [95% CI]).

Trial	n	APA	RPC	SO	DG	TOTAL
Trial 1	160	0.713 ± 0.050 [0.633, 0.793]	0.988 ± 0.161 [0.869, 1.114]	0.664 ± 0.072 [0.580, 0.750]	1.026 ± 0.044 [0.912, 1.140]	2.080 ± 0.272 [1.782, 2.414]
Trial 2	160	0.719 ± 0.129 [0.634, 0.797]	0.988 ± 0.157 [0.859, 1.123]	0.647 ± 0.074 [0.570, 0.725]	1.001 ± 0.047 [0.887, 1.120]	1.989 ± 0.296 [1.716, 2.313]
Trial 3	160	0.672 ± 0.096 [0.589, 0.753]	0.951 ± 0.182 [0.832, 1.075]	0.639 ± 0.059 [0.555, 0.719]	1.002 ± 0.110 [0.894, 1.114]	2.018 ± 0.413 [1.730, 2.335]

### 3.3 Selection of Time Window

Table 5 illustrates the predictive performance across different time windows using Trial two under the Foam + EO condition. Predictive performance stabilized after 20–30 s: the TOTAL MAE at 30 s was 1.971, exhibiting a marginal difference from 2.019 at 60 s. Balancing prediction accuracy against data acquisition efficiency, the 30-s window was selected as the input duration for subsequent experiments.

**TABLE 5:** Predictive performance across different time windows (foam + EO + trial 2, MAE ± SD [95% CI]).

Duration	n	APA	RPC	SO	DG	TOTAL
10s	160	0.682 ± 0.069 [0.604, 0.766]	1.022 ± 0.123 [0.895, 1.150]	0.644 ± 0.087 [0.562, 0.726]	0.925 ± 0.049 [0.813, 1.049]	2.116 ± 0.327 [1.821, 2.441]
20s	160	0.687 ± 0.055 [0.610, 0.767]	0.972 ± 0.139 [0.855, 1.096]	0.651 ± 0.058 [0.578, 0.727]	0.958 ± 0.076 [0.847, 1.070]	2.015 ± 0.364 [1.759, 2.304]
30s	160	0.666 ± 0.047 [0.583, 0.753]	1.011 ± 0.094 [0.887, 1.138]	0.655 ± 0.054 [0.573, 0.735]	0.958 ± 0.069 [0.848, 1.075]	1.971 ± 0.331 [1.693, 2.290]
40s	160	0.679 ± 0.077 [0.600, 0.758]	0.999 ± 0.130 [0.869, 1.136]	0.655 ± 0.045 [0.577, 0.730]	0.985 ± 0.071 [0.868, 1.110]	2.043 ± 0.383 [1.749, 2.357]
50s	160	0.681 ± 0.025 [0.599, 0.764]	0.983 ± 0.113 [0.851, 1.107]	0.639 ± 0.057 [0.556, 0.724]	0.984 ± 0.077 [0.861, 1.108]	2.032 ± 0.251 [1.758, 2.329]
60s	160	0.700 ± 0.078 [0.616, 0.786]	1.006 ± 0.143 [0.875, 1.146]	0.621 ± 0.080 [0.543, 0.702]	0.964 ± 0.057 [0.853, 1.079]	2.019 ± 0.324 [1.737, 2.342]

### 3.4 OOF predictive performance under the final selected configuration

Under this final configuration, 160 subjects had complete data and were included in the subsequent analyses. Figure 2; Figure 3 depict the scatter plots of the full-sample OOF predicted values versus true clinical scores for the Mini-BESTest total score and the four subscale scores, respectively, using the selected data configuration. The OOF Mean Absolute Error (MAE) for the total score across the entire sample was 1.979, with a Pearson correlation coefficient r = 0.758 and coefficient of determination R^2^ = 0.574. The prediction errors for the subscales were as follows: Anticipatory Postural Adjustments (APA) MAE = 0.661, r = 0.770; Reactive Postural Control (RPC) MAE = 0.982, r = 0.392; Sensory Orientation (SO) MAE = 0.647, r = 0.419; and Dynamic Gait (DG) MAE = 0.915, r = 0.693.

**FIGURE 2:**
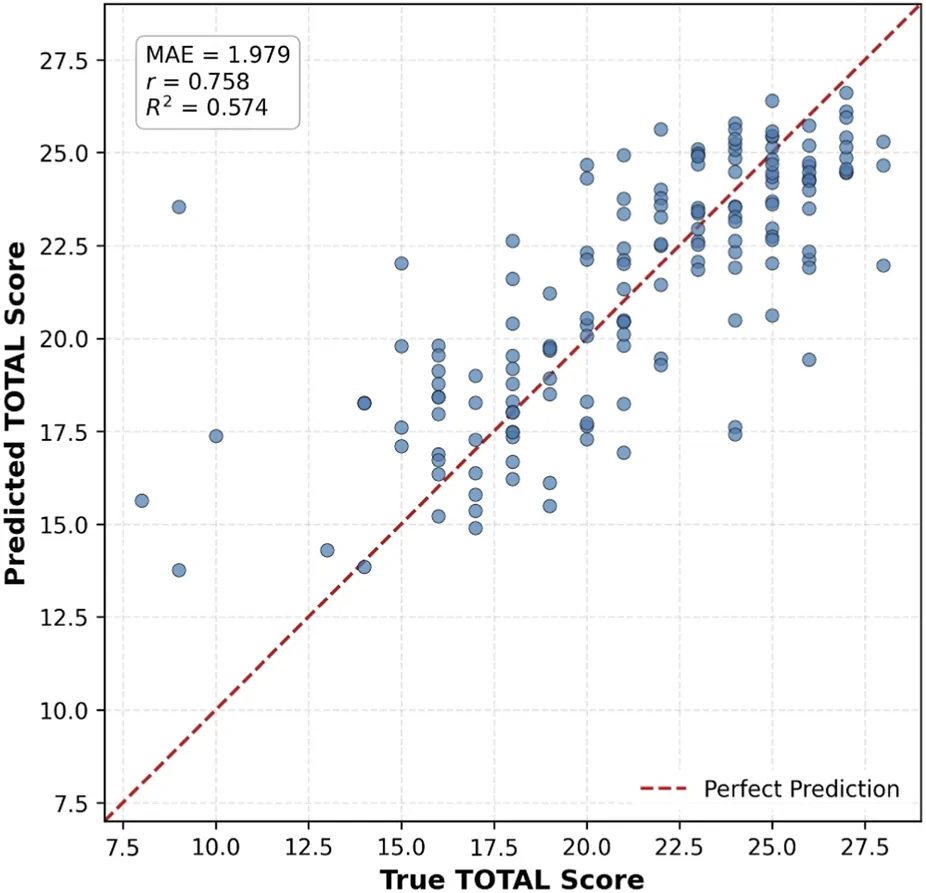
Scatter plot of OOF predictions for the Mini-BESTest total score under the final selected configuration.

**FIGURE 3:**
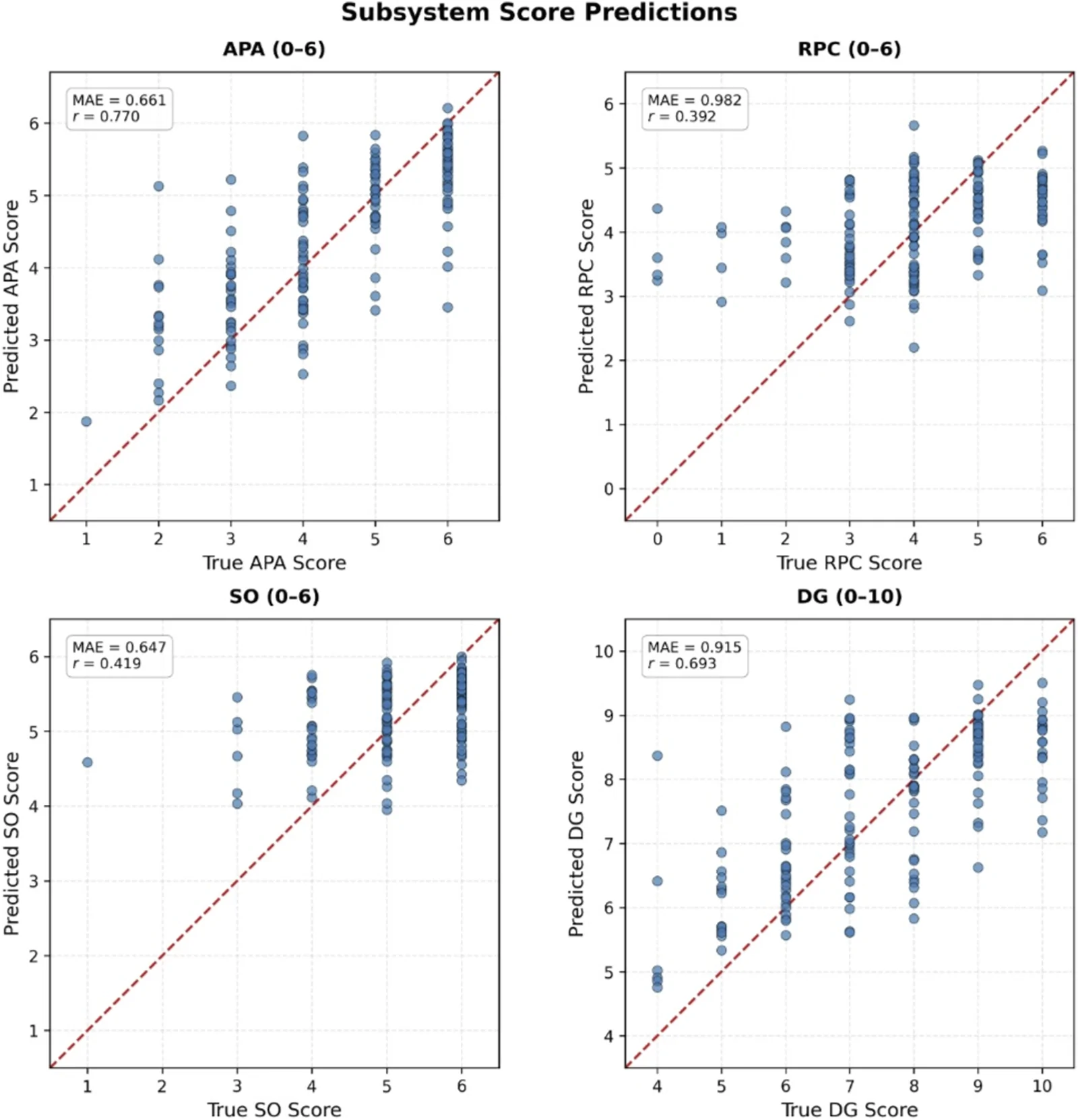
Scatter plots of OOF predictions for the four subsystems under the final selected configuration.

### 3.5 Ablation study

Table 6 presents the results of the ablation study, and Figure 4 provides a visual comparison across model variants. Numerically, removal of the metadata branch led to the largest increase in total-score MAE (from 1.971 to 2.338), with the dynamic gait subscale showing the most pronounced change (from 0.958 to 1.186). Removal of the channel attention module resulted in a smaller numerical increase in total-score MAE (to 2.034). A metadata-only model, which used the same clinical variables without COP input, achieved a total-score MAE of 2.105. Paired Wilcoxon signed-rank tests on subject-level out-of-fold total-score absolute errors, however, did not show statistically significant differences between the full model and any of the ablation variants (all p > 0.05; Table 6).

**TABLE 6:** Results of the ablation study (30s, foam + EO + trial 2, MAE ± SD [95% CI]).

Model variant	APA	RPC	SO	DG	TOTAL	p Value
Full model	0.666 ± 0.047 [0.583, 0.753]	1.011 ± 0.094 [0.887, 1.138]	0.655 ± 0.054 [0.573, 0.735]	0.958 ± 0.069 [0.848, 1.075]	1.971 ± 0.331 [1.693, 2.290]	—
−Metadata	0.764 ± 0.098 [0.666, 0.878]	0.971 ± 0.066 [0.855, 1.085]	0.630 ± 0.072 [0.552, 0.718]	1.186 ± 0.084 [1.056, 1.334]	2.338 ± 0.046 [2.012, 2.700]	0.082
−Attention	0.692 ± 0.057 [0.613, 0.774]	0.984 ± 0.107 [0.860, 1.109]	0.638 ± 0.050 [0.558, 0.712]	0.955 ± 0.091 [0.847, 1.070]	2.034 ± 0.233 [1.757, 2.328]	0.857
Metadata only	0.737 ± 0.029 [0.652, 0.815]	0.994 ± 0.166 [0.870, 1.119]	0.664 ± 0.062 [0.586, 0.745]	0.956 ± 0.106 [0.821, 1.032]	2.105 ± 0.271 [1.813, 2.412]	0.292

−Metadata = Removal of metadata branch (using only COP, signals); −Attention = Removal of channel attention module. P values were obtained from paired Wilcoxon signed-rank tests comparing subject-level out-of-fold total-score absolute errors between each ablation variant and the Full Model. None of the pairwise differences reached statistical significance (p < 0.05).

**FIGURE 4:**
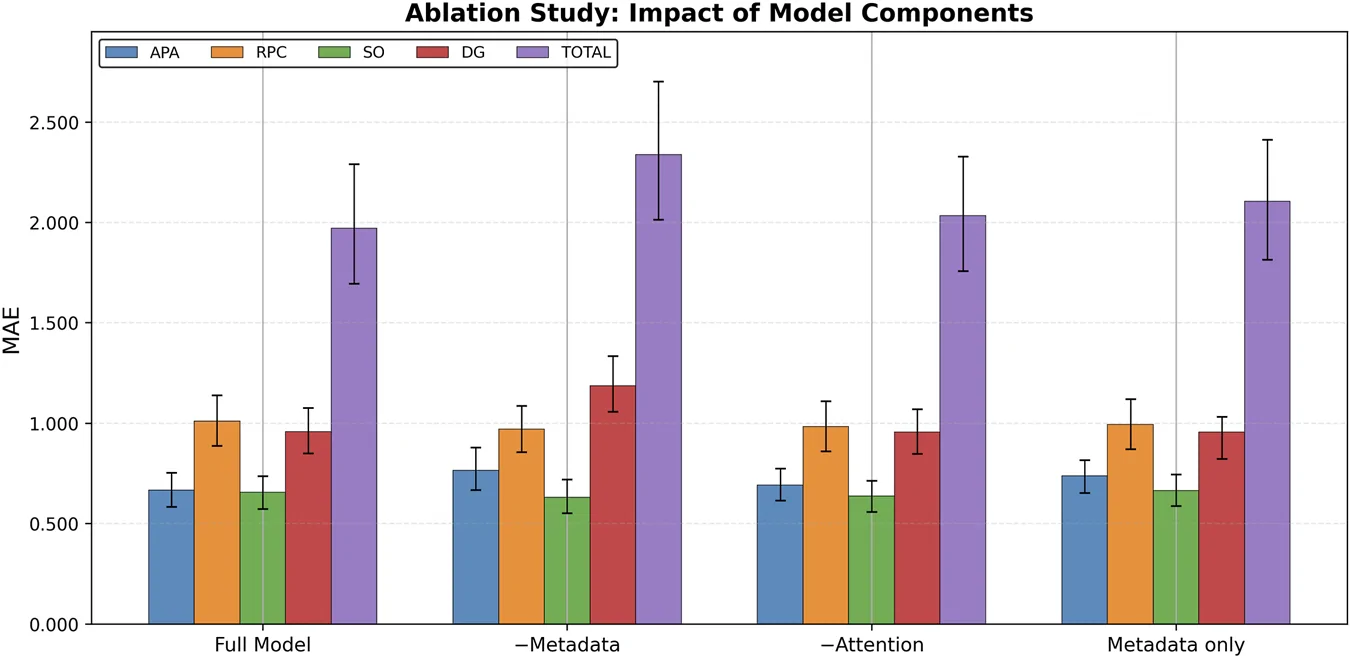
Comparative plots for the ablation study.

For additional context, the full model was compared with a simple linear regression baseline that used only the clinical metadata features. The linear regression baseline achieved a total-score MAE of 2.326, which was higher than that of the proposed full model (1.971; Table 7).

**TABLE 7:** Comparison with a simple linear regression baseline.

Model	APA	RPC	SO	DG	TOTAL
Full model	0.666 ± 0.047 [0.583, 0.753]	1.011 ± 0.094 [0.887, 1.138]	0.655 ± 0.054 [0.573, 0.735]	0.958 ± 0.069 [0.848, 1.075]	1.971 ± 0.331 [1.693, 2.290]
Linear regression (metadata only)	0.717 ± 0.020 [0.634, 0.800]	1.065 ± 0.206 [0.917, 1.183]	0.680 ± 0.073 [0.587, 0.751]	1.001 ± 0.095 [0.808, 1.032]	2.326 ± 0.275 [1.928, 2.518]

## 4 Discussion

This study proposes an end-to-end, lightweight multimodal deep learning framework that achieves out-of-fold predictions for the total Mini-BESTest score and its four subsystem scores, exclusively utilizing 30 s of quiet standing COP signals under a foam surface eyes-open condition, alongside basic clinical metadata. Under this configuration, the full-sample OOF prediction achieved a total-score MAE of 1.979, with a Pearson correlation coefficient of r = 0.758 and R^2^ = 0.574. To contextualize the clinical meaning of the observed error, the total-score MAE was compared with reported minimal detectable change (MDC) values for the Mini-BESTest. In individuals with chronic stroke, Tsang et al. (Tsang et al., 2013) reported an MDC of 3.0 points; in adults with spinal cord injury, Roy et al. (Roy et al., 2021) reported an MDC of four points, and Morooka et al. (Morooka et al., 2024) reported MDC values ranging from 3.14 to 3.84 points. The present model’s total-score MAE falls below these reported MDC estimates, suggesting that the prediction error is within the range of measurement noise associated with the clinical instrument itself. It should be noted that this does not imply that model-generated scores can directly replace clinician-administered assessments. Rather, the value of the proposed approach lies in its ability to provide a fast, objective, and repeatable estimate of balance function, potentially supporting clinicians in screening and monitoring contexts.

Compared with the prior study by Ren et al. (Ren et al., 2020), which predicted Mini-BESTest scores from the same public dataset, the present work differs in both modeling approach and data requirements. Ren et al. (Ren et al., 2020) extracted 224 handcrafted COP features and trained traditional regressors, whereas this study used an end-to-end deep learning model with raw COP signals and clinical metadata as input. In addition, the present model used only 30 s of quiet standing from a single condition (Foam + EO), while the prior approach used COP data from all four standing conditions. Despite the reduction in input data and the removal of handcrafted feature engineering, the total-score MAE reported here (1.971) is numerically lower than that previously reported (2.658). This does not by itself demonstrate that the proposed framework is superior, since the two studies also differed in other implementation details. Nevertheless, the results suggest that end-to-end learning may provide a more direct and potentially more data-efficient pathway for automated balance assessment. This implication may be useful for future research aiming to simplify data collection protocols and reduce reliance on expert-designed features.

An observable phenomenon in this study is that COP patterns may differ considerably across stance conditions, and the performance of a model trained on mixed multi-condition data (All Conditions) proved inferior to that of a single condition. This aligns with the intuitive understanding of underlying mechanistic differences in balance control across conditions—different conditions may challenge distinct aspects of the balance control system, and variations in COP signal patterns likely reflect the differing degrees of subsystem engagement. The relatively better performance achieved under the foam condition may be attributed to the foam surface disrupting plantar somatosensory input, thereby compelling the subject to rely more heavily on vestibular and visual information to maintain equilibrium. Consequently, the resulting COP signals may encapsulate richer information pertinent to balance control.

The ablation results showed a numerical trend in which removal of the clinical metadata branch led to the largest increase in total-score MAE, with the dynamic gait subscale showing the most pronounced change. This pattern suggests that while COP signals contain postural control-related information, they may not fully cover gait-related balance functions when used alone. Clinical metadata may therefore provide complementary information to COP signals. It should be noted that paired tests based on subject-level out-of-fold errors did not show these differences to be statistically significant. Age, cognitive function (TMT), and fear of falling (FES) are examples of clinical metadata that may reflect the overall functional status and compensatory strategies of the subjects (Meseguer-Henarejos et al., 2025). It should be emphasized that the clinical metadata utilized in this study constitute routine clinical assessments typically acquired during initial patient visits or annual examinations (Santos and Duarte, 2016). In clinical practice, these data can be extracted directly from electronic health records, eliminating the need for repeated acquisition during every balance assessment. For scenarios with missing metadata, future work could explore model variants relying solely on COP signals (akin to the -Meta version in our ablation study), despite a potential decrease in predictive precision. This finding also offers a viable direction for future model enhancements: incorporating additional clinical variables relevant to balance control could further improve predictive accuracy.

At the subsystem prediction level, performance varied across assessment dimensions. Sensory Orientation (SO) consistently showed relatively small absolute prediction errors, which may reflect that the foam surface with eyes open condition itself targets sensory integration, and COP signals during this task are associated with the corresponding subscale scores. Correlation analyses indicated that Anticipatory Postural Adjustments (APA, r = 0.770) and Dynamic Gait (DG, r = 0.693) showed relatively higher linear relationships with the predicted scores. It should be noted that these predictions reflect statistical associations among clinical scores rather than direct measurement of the underlying dynamic or reactive balance functions. Reactive Postural Control (RPC) showed the largest prediction error and the lowest correlation (r = 0.392), consistent with the fact that quiet standing without external perturbation provides limited information about this subsystem.

The model architecture proposed in this study contains approximately 55 K parameters, which places it within the lightweight category. As a reference, deep learning models commonly used in this field, such as the ResNet-18 backbone employed by Choi et al. (Choi et al., 2024), typically contain parameters on the order of millions. While maintaining comparable predictive accuracy, the current model reduces the parameter count by approximately two orders of magnitude, suggesting lower computational overhead. This property may make the model a candidate for future deployment on portable force plates, embedded systems, or mobile devices in cost- and power-sensitive settings, such as community health screenings, routine nursing home assessments, or remote rehabilitation monitoring (Iqbal et al., 2024; Baig et al., 2026). However, no inference latency, memory consumption, or energy measurements were performed in the present study, and the model has not yet been evaluated on an actual portable device or in prospective remote or community settings. Progressing from these findings to practical deployment will therefore require further engineering optimization and device-level validation.

Several limitations are acknowledged in this study. First, the dataset is a convenience sample derived from a single research center and represents a community-mixed population rather than a disease-specific neurological cohort. The age distribution is also somewhat uneven (concentrated primarily in the 20–40 and 60–90 age ranges). These factors may affect the generalizability of the model to broader or clinically defined populations. Second, five subjects failed to complete all trials; although handled according to available trials, this could introduce minor evaluation bias. Third, this study exclusively utilized COP signals and demographic/cognitive/psychological metadata, omitting other physiological signals potentially associated with balance control (e.g., electromyography or kinematic data). Fourth, the data configuration selection and final performance evaluation were executed within the same cross-validation framework. The reported MAE values may, to some extent, reflect an optimized outcome for this specific dataset; the model’s performance requires prospective validation on an independent external dataset. Furthermore, given the limited interpretability intrinsic to deep learning models, future work could integrate methodologies such as attention visualization and SHAP to enhance clinical acceptability.

## 5 Conclusion

This study develops an end-to-end, lightweight multimodal deep learning framework capable of predicting the total Mini-BESTest score and its four subsystem scores using 30 s of quiet standing COP signals under a foam surface eyes-open condition, combined with basic clinical metadata. An appropriate data configuration was identified through a series of comparative experiments, and the contributions of individual components were assessed via ablation studies. Performance estimates were obtained through an OOF procedure, in which predictions for each subject were generated from models that had not seen that subject during training. This lightweight framework (with approximately 55 K parameters) demonstrates potential for automated balance assessment. Its applicability to community screening and remote rehabilitation monitoring requires further validation in the intended populations.

## Data Availability

Publicly available datasets were analyzed in this study. This data can be found here: PhysioNet at https://doi.org/10.13026/C2WW2W and on Figshare at https://doi.org/10.6084/m9.figshare.3394432 (dataset name: ‘A public data set of human balance evaluations’ by Santos and Duarte, 2016).
